# Global Metabolic Reconstruction and Metabolic Gene Evolution in the Cattle Genome

**DOI:** 10.1371/journal.pone.0150974

**Published:** 2016-03-18

**Authors:** Woonsu Kim, Hyesun Park, Seongwon Seo

**Affiliations:** Department of Animal Biosystem Sciences, Chungnam National University, Daejeon, Republic of Korea; University of Lausanne, SWITZERLAND

## Abstract

The sequence of cattle genome provided a valuable opportunity to systematically link genetic and metabolic traits of cattle. The objectives of this study were 1) to reconstruct genome-scale cattle-specific metabolic pathways based on the most recent and updated cattle genome build and 2) to identify duplicated metabolic genes in the cattle genome for better understanding of metabolic adaptations in cattle. A bioinformatic pipeline of an organism for amalgamating genomic annotations from multiple sources was updated. Using this, an amalgamated cattle genome database based on UMD_3.1, was created. The amalgamated cattle genome database is composed of a total of 33,292 genes: 19,123 consensus genes between NCBI and Ensembl databases, 8,410 and 5,493 genes only found in NCBI or Ensembl, respectively, and 266 genes from NCBI scaffolds. A metabolic reconstruction of the cattle genome and cattle pathway genome database (PGDB) was also developed using Pathway Tools, followed by an intensive manual curation. The manual curation filled or revised 68 pathway holes, deleted 36 metabolic pathways, and added 23 metabolic pathways. Consequently, the curated cattle PGDB contains 304 metabolic pathways, 2,460 reactions including 2,371 enzymatic reactions, and 4,012 enzymes. Furthermore, this study identified eight duplicated genes in 12 metabolic pathways in the cattle genome compared to human and mouse. Some of these duplicated genes are related with specific hormone biosynthesis and detoxifications. The updated genome-scale metabolic reconstruction is a useful tool for understanding biology and metabolic characteristics in cattle. There has been significant improvements in the quality of cattle genome annotations and the MetaCyc database. The duplicated metabolic genes in the cattle genome compared to human and mouse implies evolutionary changes in the cattle genome and provides a useful information for further research on understanding metabolic adaptations of cattle.

## Introduction

One of the most important purposes of livestock is to supply high-quality food (energy, protein and fat) for humans [1]. Ruminants (e.g., cattle, sheep and goat) are highly efficient in utilizing nutrients from plant-origin feeds compared to monogastric animals due to its specialized organ, rumen [2]. Among ruminants, cattle (*B*. *taurus* and *B*. *indicus*) produce meat and milk, which contribute 15% of the total protein consumed by humans in the world [3]. Therefore, there have been many attempts to better understand the unique features of ruminant metabolism for increasing production, feed and growth efficiency and disease resistance. Additionally the unique genomic features make the cattle genome a great resource for investigating the evolution of the mammalian genomes [4]. In this regard, the cattle genome had been sequenced and annotated, which provided a valuable opportunity to systematically link genetic and metabolic traits of cattle.

One of the ways to link genetic and metabolic traits of an organism is to perform a genome-scale model via metabolic reconstruction [5]. To reconstruct a genome-scale metabolic model, high-quality genomic information with structural and functional annotations is required, which can be obtained from web-based biological databases. The amalgamation of genomic information from the biological databases is thus a necessary step for metabolic reconstruction and the subsequent construction of genome-scale model. Previously an amalgamated cattle genome database was developed and metabolic pathways of the cattle genome was reconstructed based on the cattle genome build Btau_3.1, named as CattleCyc [6]. CattleCyc (http://168.188.16.73:8080/CATTLE) is the first pathway genome database (PGDB) developed in livestock animals, generated using Pathway Tools and the BioCyc platform [7], followed by manual curation. The CattleCyc provides a platform to identify metabolic conservation and differences among different organisms. Although there were only a few differences in core metabolic pathways between the human and cattle genomes [6], comparison of reconstructed metabolic pathways between two organisms revealed some unique features of biology in cattle [8]. After CattleCyc was developed, a new and better reference genome build has been assembled and annotated (i.e. UMD_3.1). The cattle genome build UMD_3.1, a reassembly of the whole genome shotgun sequences used for the build Btau_3.1 (chromosome 1–29 and X), provides more and better gene models and annotations of the cattle genome, compared to the previous build [9]. An update of CattleCyc based on the new build would, thus, provide an opportunity for better understanding of the biology of cattle.

Moreover, although metabolic gene deletions in the cattle genome compared with the mouse and human genomes were identified previously [8], metabolic gene duplications in the cattle genome have not been studied. The presence and/or absence of metabolic genes in a species can result in various behavior of metabolic responses, considering interconnectivity and complexity of biological system [6]. Gene duplication is believed to play an important role in speciation during the course of evolution [10]. Gene duplication may not be an essential process for a species to survive; however, it is important for the species to maintain or expand its ecological niches [11]. Among mammalian species, gene duplications are commonly observed in the genes associated with recognition of environmental signals, innate immunity, detoxification, olfaction, and so on [11]. Like the metabolic gene deletions in the cattle genome compared to the human and mouse genomes as previously reported [8], identification of duplicated metabolic genes in the cattle genome may also provide biological insights of metabolic adaptations occurred in cattle.

Therefore, the objective of this study was to reconstruct genome-scale cattle-specific metabolic pathways based on the updated reference cattle genome build UMD_3.1 and compare the newly reconstructed cattle metabolic pathways with those in CattleCyc (based on Btau_3.1). This study also identified the cattle-specific gene duplications of metabolic genes compared to the human and mouse genomes.

## Methods

**Fig 1:**
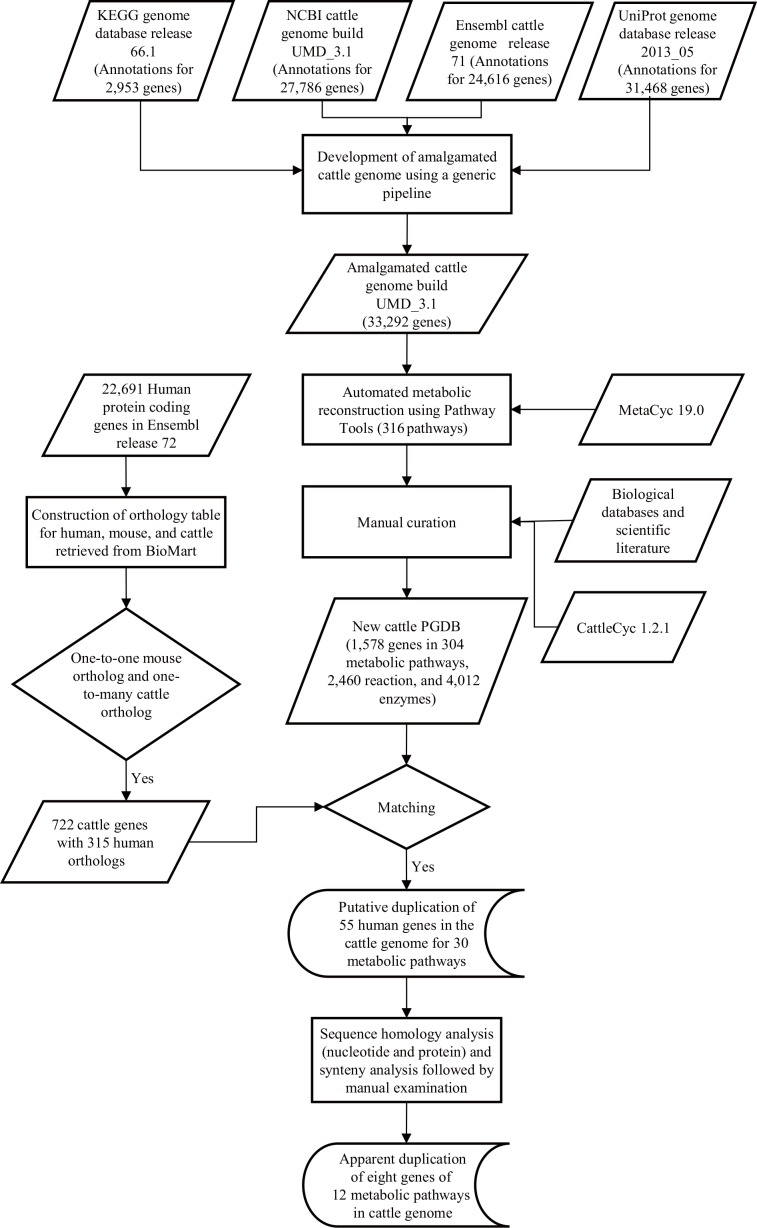
The experimental approach used for the reconstruction of a new cattle PGDB and identification of metabolic gene duplications in the cattle genome. Solid and dash arrows represent data and information flows, respectively.

The general scheme of the metabolism-centered approach used for reconstruction of metabolic pathways and identification of duplicated metabolic genes in the cattle genome is represented in Fig 1.

### The amalgamated cattle genome database

A revised amalgamation pipeline was developed on the basis of the previous one as described by Seo and Lewin [6]. Briefly, the amalgamation pipeline automatically downloads the information of gene, protein, enzyme, reactions, and metabolic pathways of an organism from various biological databases: NCBI [12], Ensembl [13], KEGG [14] and UniProt database [15]. For this study, the NCBI cattle reference build UMD_3.1 using Entrez-Gene [16], the Ensembl release 71 using Biomart [17], the UniProt knowledgebase release 2013_05, the Kyoto Encyclopedia of Genes and Genomes (KEGG) Genome Database release 66.1 were retrieved via FTP on May 10, 2013. The NCBI and Ensembl genome databases were separately used for the basal structural and functional annotations for the cattle genome. The NCBI- and Ensembl-based comprehensive cattle genome databases were independently constructed by incorporating all the known protein, enzyme, enzymatic reaction and metabolic pathway information obtained from UniProt and KEGG by matching the ID, names and synonyms of gene and gene products, and other cross-referenced identification. The above NCBI- and Ensembl-based comprehensive cattle genome databases were then integrated for removing redundancy, and an amalgamated genome database was generated. During the integration, a sequential matching process was performed for all gene pairs that shared a common (partial or complete) chromosomal location between two genome databases, including those on unassigned contigs. The two genes were matched based on the following criteria sequentially: if two genes have 1) exactly the same gene coordinates, or have common 2) gene name or synonyms, 3) function names, 4) unigene ID, 5) cross-referenced gene ID, 6) protein accessions, or 7) the Enzyme Commission (EC) numbers. In the updated pipeline, unlike the previous one, Unigene ID and protein accession were added to the matching criteria and genes are matched regardless of different strand or biotypes (e.g., protein coding gene, pseudogene, ncRNA). These updates have increased the number of consensus genes between the NCBI- and Ensembl-based comprehensive cattle genome databases even though additional manual examination is needed to eliminate false-positives.

### Genome-scale metabolic reconstruction of the cattle genome

Genome-scale reconstruction of metabolic pathways from the amalgamated cattle genome database was performed using the PathoLogic algorithm in the Pathway Tools software version 19.0 [18]. Pathway Tools computationally reconstructs organism-specific metabolic pathways and generates a new PGDB that contains the genes, proteins, biochemical reactions and predicted metabolic pathways [18]. Using the PathoLogic algorithm, the EC numbers, the gene-product names (i.e. protein names) and the GO (Gene Ontology) ID information in the amalgamated cattle genome database were matched with those of enzymes in a specific metabolic pathway stored in MetaCyc, a manually curated database containing 2,260 pathways from 2,072 different organisms [19].

The initial automated reconstruction identified 316 metabolic pathways in the amalgamated cattle genome based on UMD_3.1. The initial cattle-specific PGDB intrinsically contained some errors such as pathway holes in which the organism-specific enzyme has not yet been identified and false-positive or false-negative pathways. Therefore, an intensive manual curation was performed to revise improper enzymatic reactions and metabolic pathways using various approaches including literature reviews, database searches, and comparison of metabolic pathways with other organisms.

A metabolic pathway was deleted in the new cattle-specific PGDB if 1) redundant pathways existed (e.g. 2-methylbutyrate biosynthesis was redundant with 2-methylbutyrate biosynthesis mammals), 2) either the input or the output of the pathway was not present in any mammal (e.g. peptidoglycan biosynthesis III), 3) neither enzyme activity was reported nor homologs were identified in any mammal, and an alternative pathway exists with strong biochemical evidence (e.g. lysine degradation V). When representation of a mammalian pathway in MetaCyc was not adequate, the pathway was modified based on information from the literature, KEGG, and Brenda [20]. The proteins of pathway holes for which no gene was identified were searched for in the cattle genome and non-redundant protein databases using TBLASTN and BLASTP [21], respectively. The thresholds used for identification of the cattle ortholog of a mammalian protein were 80% coverage and 70% identity which adopted in the Ensembl gene annotation [22]. In additional, orthologs were assigned if the best BLAST hit included >50% and exactly matched >90% of the query protein sequence as described by Seo and Lewin [6].

The metabolic pathways in the new cattle PGDB was also compared with those in the CattleCyc (version 1.2.1) in the BioCyc databases [23]. With this, not only the curation of the new cattle PGDB based on previously well-curated PGDB for the same organism was possible, but also improvement in the cattle genome annotations and the MetaCyc database could be evaluated.

The CattleCyc (version 1.2.1) and the new cattle PGDB are freely accessible via http://168.188.16.73:8080/CATTLE and http://168.188.16.73:8080/TAURUS, respectively. Both PGDB are also deposited into the Figshare data repository (https://dx.doi.org/10.6084/m9.figshare.3082039).

### Identification of gene duplication

To identify cattle-specific duplicated genes, an orthology table for human, mouse and cattle generated by BioMart (http://www.biomart.org) was used. First we obtained a list of human protein coding genes having one-to-one mouse and one-to-many cattle orthologs and a list of mouse protein coding genes having one-to-one human and one-to-many cattle orthologs from the Ensembl release 72. By comparing these list with the metabolic genes in the new cattle-specific PGDB, putative duplicated metabolic genes in the cattle genome were identified.

These putative duplicated genes underwent multiple levels of evaluation for determining whether these putative duplication were due to cattle-specific gene duplication or assembly error. We conducted comprehensive analysis of sequence similarity at the nucleotide and protein levels and synteny of mammals, followed by intensive manual examination. For confirming gene duplications for cattle, we used information from web-based genome databases (NCBI, Ensembl, Biomart, UCSC genome browser and etc.) and literature. The putative duplicated genes were ranked by the following criteria: A) supported by the information of nucleotide chain, expressed sequence tag (EST), evidence for expression (i.e., mRNA sequence or protein evidence), and completeness of the sequencing and assembly, B) supported by the evidence of nucleotide chain, EST, and mRNA or protein evidence, but with a possibility of assembly error (i.e. scaffold gaps), C) supported by the evidence of nucleotide chain, but lacking information of either EST or mRNA (or protein) evidence and with a possibility of assembly error, D) supported by the evidence of nucleotide chain, but lacking both EST and mRNA (or protein) evidence and with a possibility of assembly error. The identified cattle-specific duplicated genes were also analyzed for their biological and metabolic functions using the amalgamated functional annotations in the new cattle PGDB.

## Results

### The amalgamated cattle genome databases based on UMD_3.1

Total numbers of 27,786 and 24,616 genes were contained in the NCBI and Ensembl cattle genome database based on UMD_3.1. Through the matching process, 2,467, 5,742, 10,009, 11,874, 16,317, 16,423 and 1,255 consensus genes between the NCBI and Ensembl cattle genomes were non-exclusively identified based on the criteria of gene coordinate, gene name (or synonym), function name, unigene ID, cross-referenced gene ID, protein accession, or EC numbers, respectively (Table 1).

**Table 1:** The number of consensus cattle gene pairs in the NCBI and Ensembl cattle genome databases.

	Number of matched pairs		
		Sequential unique matches	
Type of match	Non-exclusive matches	UMD 3.1	Btau 3.1
Gene coordinates	2,467	5,295	2,109
Gene name	5,742	5,963	5,187
Function name	10,009	4,693	71
Unigene ID	11,874	1,398	-
Cross-reference gene ID	16,317	1,511	8,800
Protein accessions	16,423	262	-
EC numbers	1,255	1	6
Manually matched*			27
Total	64,087	19,123	16,200

*Manually matched after manual evaluation. Those pairs were not matched because the genes were classified with different gene type or strand in the NCBI and Ensembl gene models.

The sequential one-to-one matching between the NCBI and Ensembl cattle genome annotations, a total of 19,123 consensus gene models were found. Among these, 854 genes were inconsistently annotated for gene type, coding strand, or both between the NCBI and Ensembl genome databases: 821, 21 and 12 genes were differently annotated in gene type, coding strand, or both, respectively. In the consensus gene set 5,295, 5,963, 4,693, 1,398, 1,511, 262 and one genes were sequentially matched to have common gene coordinates, gene name or synonyms, function names, unigene ID, cross-referenced gene ID, protein accessions and EC numbers, respectively (Table 1). Compared to the previous amalgamated cattle genome build Btau_3.1 in CattleCyc, 2,923 more genes were identified as a consensus gene set in this study (Table 1). It implied that there has been an improvement in the amalgamation pipeline, consistency of cattle genome annotations in UMD_3.1, or both.

As a result, the amalgamated cattle genome database, based on the reference cattle genome build UMD_3.1, contained a total of 33,292 genes consisting of 19,123 consensus genes, 8,410 and 5,493 genes only found in NCBI or Ensembl, respectively, and additional 266 genes from NCBI scaffolds genes (Table 2).

**Table 2:** Distribution of genes in the amalgamated cattle genome database according to the original data sources.

	Number of genes	
Type of match	Btau_3.1	UMD_3.1
Consensus	16,200	19,123
NCBI build only	12,287	8,410
Ensembl build only	8,932	5,493
NCBI genome scaffolds*	245	266
Total	37,664	33,292

*Scaffolds not in the current genome assembly but included in the NCBI reference genome build

### Reconstruction of the cattle-specific metabolic pathways

The initial cattle PGDB automatically constructed 316 metabolic pathways using the PathoLogic algorithm. The initial automated reconstruction identified 2,460 reactions, including 2,371 enzymatic, 73 spontaneous and 16 transport reactions, and 1,489 compounds (Table 3). An enzymatic reaction is defined as a chemical reaction that involves a single enzyme or an enzyme complex but does not mediate molecular transport. Among the enzymatic reactions, a total of 202 pathway holes were identified in 93 metabolic pathways, which accounted for 19% of the total reactions in the reconstructed pathways. During the manual curation, 68 pathway holes were filled; the organism-specific enzyme was identified. Through this filling-hole process a total of 57 metabolic pathways were revised; any error in enzymatic reactions was corrected, and genes encoding specific enzymes were assigned (see more details in S1 Table). Some redundant and non-mammalian metabolic pathways were deleted during manual curation. The deletions include 36 pathways: 11 and 25 due to redundancy and non-mammals pathways, respectively. For example, the 2-methylbutyrate biosynthesis pathway was deleted because it is redundant with 2-methylbutyrate biosynthesis pathway mammals. Another pathway, Peptidoglycan biosynthesis III, which does not exist in mammals, was also eliminated from the reconstruction.

**Table 3:** Comparison of cattle-specific pathway genome database (PGDB).

	New cattle PGDB^†^				
Database statistics	Initial	Curated			CattleCyc^‡^
Metabolic pathways	316	304			218
Enzymatic reactions	2,371	2,371			1,439
Enzymes	4,012	4,012			1,544
Compounds	1,489	1,489			1,006
**Pathway holes**					
Number of pathways holes	202	134			134
Percentage*	19%	13%			17%
Pathway with no holes	219	237			154
Pathway with 1 hole	42	32			38
Pathway with 2 holes	22	19			13
Pathway with 3 holes	17	7			2
Pathway with 4 holes	3	2			7
Pathway with >4 holes	9	7			7
Total pathway with holes	93	67			67

^†^Cattle specific pathway genome database constructed based on UMD_3.1

^‡^CattleCyc constructed based on Btau_3.1

*Pathway holes as percentage total reactions in pathways

**Fig 2:**
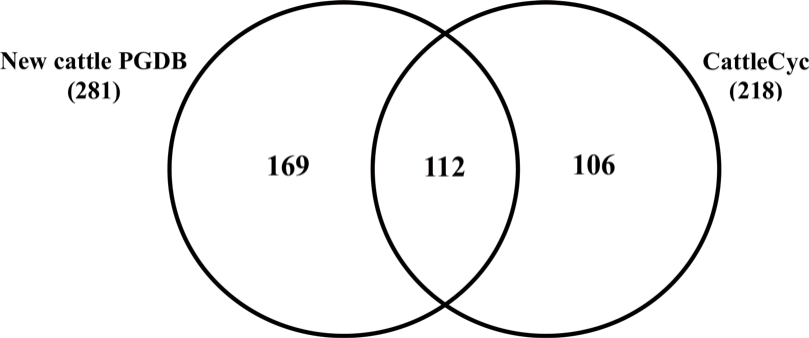
Comparison of cattle-specific pathway genome database (PGDB) based on UMD_3.1 with the previous version of cattle PGDB based on Btau_3.1 (CattleCyc 1.2.1; http://biocyc.org)

In addition, the metabolic pathways in the new cattle PGDB were compared with those in the CattleCyc, a previously developed cattle PGDB based on the genome build Btau_3.1. The CattleCyc is publicly available and can be downloaded from the BioCyc registry. There were 169 and 104 pathways, contained only in the new cattle PGDB or in the CattleCyc, respectively (Fig 2). Among the 169 pathways included only in the new cattle PGDB, 110 and 59 pathways were added because of improvements in the MetaCyc and the cattle genome assembly, respectively. Whereas, 106 pathways in the CattleCyc were not included in the initial automated metabolic reconstruction due to MetaCyc updates (53), insufficient evidence for incorporation (14), and errors in the CattleCyc (16) (see more details in S2 Table). The remaining 23 metabolic pathways in the CattleCyc were added in the new cattle PGDB. As a result, the cattle PGDB based on the cattle genome build UMD_3.1 contained 304 metabolic pathways (Table 3).

### Duplicated metabolic genes in the cattle genome compared to human and mouse

The cattle-specific duplicated genes were identified using an orthology table for human, mouse and cattle. The total protein coding genes in human, mouse and cattle genome were 22,691, 22,709 and 19,994, respectively. A total of 315 out of 22,691 human protein coding genes in the Ensembl release 72 had one-to-one mouse and one-to-many cattle orthologs. Among these, 55 genes were the metabolic genes in the cattle metabolic reconstruction, involved in 30 metabolic pathways. Those genes underwent comprehensive analysis of sequence similarity at the nucleotide and protein levels and synteny analysis, followed by intensive manual examination on the basis of our criteria. The total number of apparently duplicated metabolic genes in the cattle genome was eight being involved in 12 core metabolic pathways (Table 4).

**Table 4:** List of cattle-specific duplicated metabolic genes compared to human and mouse.

Gene name	Chr	Start	End	Description	Pathway
*AANAT*	19	55904213	55905449	Arylalkylamine N-acetyltransferase	Serotonin and melatonin biosynthesis
	19	55917031	55918872		
*ACOT4*	10	85431609	85435220	Acyl-CoA thioesterase 4	Acetyl-CoA hydrolysis, oleate biosynthesis II (animals)
	10	85375286	85380537		
*ADK*	15	35836626	35837711	Adenosine kinase	Adenine and adenosine salvage VI
	28	30215525	30732466		
*BPGM*	4	99223160	99254542	2,3-biphosphoglycerate mutase	Glycolysis/gluconeogenesis, Rapoport-Luebering glycolytic shunt
	10	78463269	78464048		
*HEXB*	20	6721327	6753670	Hexosaminidase B	chondroitin sulfate degradation (metazoa), dermatan sulfate degradation (metazoa)
	20	6760133	6794235		
*GSTO1*	26	25060114	25074160	Glutathione S- transferase omega 1	Arsenate detoxification I (glutaredoxin), glutathione-mediated detoxification
	26	25088448	25097722		
*NDUFB4*	1	65922482	65928620	NADH hydrogenase (ubiquinone) 1 beta sub complex, 4, 15kda	Aerobic respiration (cytochrome c)
	21	13421643	13422116		
*SOD1*	1	3113948	3122613	Superoxide dismutase 1, soluble	Superoxide radicals degradation
	13	51930067	51930888		

**Fig 3:**
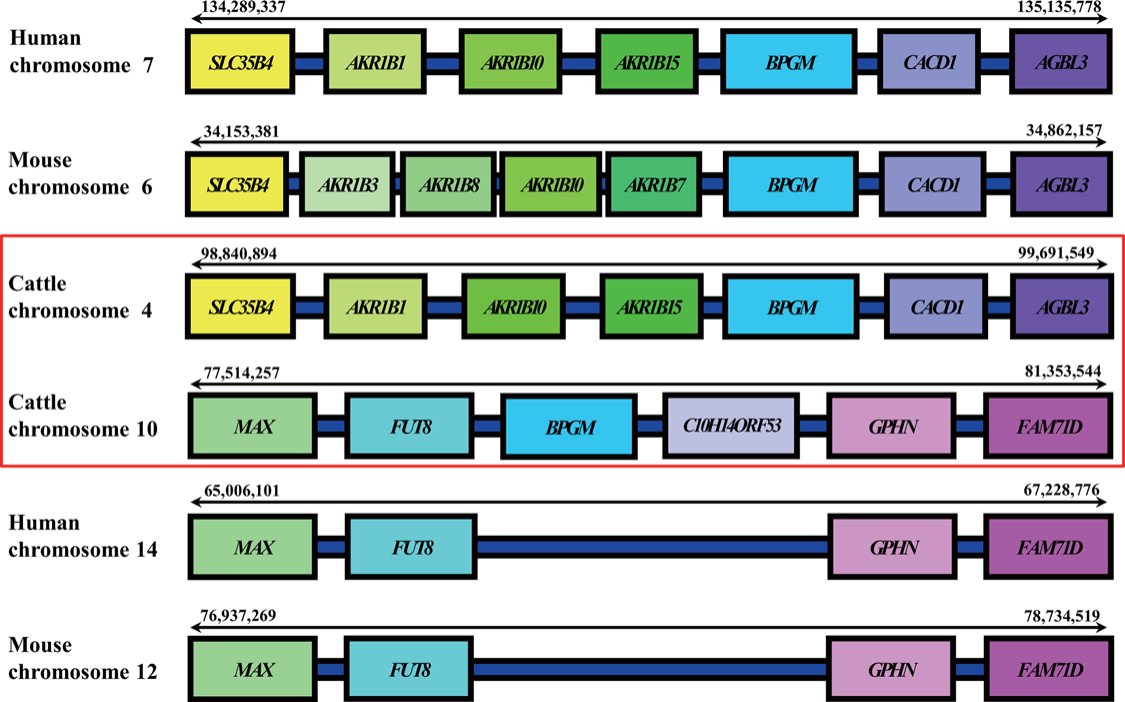
Gene duplication of BPGM in the cattle genome. Up- and down-stream flanking genes of each duplicated BPGM are shown.

Unfortunately, none of the genes was classified as ‘A’ based on our criteria. Two genes, *HEXB* and *GSTO1*, have been annotated with EST and protein accession so they were assigned in ‘B’ category. Five genes (i.e., *AANAT*, *ACOT4*, *ADK*, *NDUFB4*, and *SDO1*) had at least one of either EST or protein evidence were classified as ‘C’. The *BPGM* gene was ranked ‘D’ according to our criteria due to lack of expression evidence. Nevertheless, *BPGM* showed an interesting feature based on synteny analysis among human, mouse and cattle (Fig 3). The *BPGM* in the cattle genome is located in two different genomic locations; chromosome 4 and chromosome 10. The order of up- and down flanking genes of the one in chromosome 4 were conserved in both human and mouse genomes. In contrast, when comparing the evolutionary synteny block among three organisms, the genomic region for the cattle *BPGM* on chromosome 10 was not found in both human and mouse genome although the up- and down-flanking genes were conserved (Fig 3). It indicated that the *BPGM* gene was possibly duplicated in the cattle genome.

These duplicated metabolic genes in the cattle genome involve in 12 core metabolic pathways, such as hormone biosynthesis, fatty acid in lipid biosynthesis, nucleosides and nucleotides biosynthesis, generation of precursor metabolites and energy, carbohydrate biosynthesis, metabolic regulators biosynthesis, degradation/utilization/assimilation, and detoxification (Table 4).

## Discussion

### Development of the Amalgamated Cattle Genome Database

It is important to note that the genome research is heavily dependent on the quality of genome annotations. For the first time in the cattle genome, Seo and Lewin [6] tried to resolve the discrepancy in the annotations of the cattle genome between the NCBI and Ensembl databases. They reported that in some cases the same genes have different gene type and coordinate strand in NCBI and Ensembl databases. Based on this, we developed a new pipeline and improved the matching process for increasing quality of cattle genome annotations. The matching criteria were modified to consider the discrepancy gene type and coordinate strand in two databases, and to have new criteria with unigene ID and protein accession.

Consequently, the new amalgamated cattle genome database contained 2,950 more consensus genes than Btau_3.1. This may be possible because the quality of cattle genome annotations in the public databases has been improved and/or the new amalgamation pipeline is more efficient than the previous one. During the matching process, nonetheless, we also found that some discrepancy in functional annotation of genes remained in NCBI and Ensembl databases. This inconsistency among genome databases suggests that researchers should be cautious for gathering biological information from the databases, and amalgamation of annotations from different biological databases is critical.

### Reconstruction of the cattle-specific metabolic pathways

We reconstructed cattle-specific metabolic pathways for better understanding of underlying biological insights and metabolic traits in cattle. The reconstruction process was done using Pathway Tools software equipped with the pathologic algorithm. The Pathway Tools has several advantages for reconstruction of genome-scale metabolic network. First, the metabolic pathways of an organism can be automatically reconstructed on the basis of a comprehensively curated biological pathway database, MetaCyc, by the pathologic algorithm. Second, the reconstructed organism-specific PGDB can easily be used for comparative analysis of metabolic network among different species. Third, various genomic data, such as transcriptomic, proteomic, metabolomic and reaction flux, can be integrated into the reconstructed metabolic pathways by the Pathway Tools Omics Viewer. Fourth, the PGDB generated from MetaCyc have low errors such as unbalanced reactions and orphan and dead-end metabolites than those generated from KEGG, and can be easily used for flux balance analysis [24].

Nonetheless, the initial automatically reconstructed PGDB needs intensively manual curation process because some false-positive and false-negative pathways contain in the initial PGDB. This is mainly due to the fact that pathologic algorithm was designed to import as many candidate metabolic pathways from MetaCyc which is mainly composed of bacteria and plant metabolic pathways [19]. Consequently, a large proportion of predicted metabolic pathways are redundant and/or are not mammalian pathways, and they need to be deleted from the automated reconstruction. CattleCyc also deleted and modified 53% of the initial automated reconstruction metabolic pathways by manual curation [6].

The initial reconstruction in this study, however, contained less false-positive and -negative pathways than the one when developing CattleCyc, mainly because the well-curated CattleCyc as well as MetaCyc was also used as a base for the new metabolic reconstruction. Still, the unknown enzyme, enzymatic reactions and enzyme encoded protein in metabolic pathway, called pathway holes, needed to be filled with correct annotations for increasing the quality of the cattle PGDB. For example, in the triacylglycerol degradation pathway, the pathway hole for the reaction of EC 3.1.1.34 was modified to EC 3.1.1.79 and filled with *LIPE* (hormone-sensitive lipase). The reaction of EC 3.1.1.- in this pathway was also replaced by EC 3.1.1.3. In addition, 11 redundant pathways, and 25 bacteria or plant-specific pathways were deleted from the initial reconstruction.

Comparative metabolic analysis indicated that the new cattle PGDB was better than CattleCyc. This became possible primarily due to the improvement in the cattle genome build and in the MetaCyc database. For example, the ‘glucuronate degradation’ pathway did not exist in the CattleCyc because L-gulonate 3-dehydrogenase (EC 1.1.1.45) encoded by *CRYL1* was missing in the previous cattle genome build; however, it was annotated in the current build and thus included in the new cattle PGDB. The primary reason that the 83 metabolic pathways exist in the CattleCyc were not initially included in the new cattle PGDB was the updates of MetaCyc. For example, the ‘pyruvate oxidation’ pathway was included in the CattleCyc. This pathway oxidatively catalyzes pyruvate to acetate and CO_2_ by pyruvate:quinone oxidoreductase (EC 1.2.2.2) [25]. Upon the recent update of MetaCyc, this enzyme has been replaced by pyruvate dehydrogenase (quinone) (EC 1.2.5.1); however, this enzyme exists only in bacteria [26], and the enzyme activity has been not found in mammals. The pathway was thus deleted in the new cattle PGDB. On the other hand, the 14 metabolic pathways in CattleCyc were found to be false-positives due to errors of the previous cattle genome build (Btau_3.1).

These results indicated that a comparative analysis of metabolic pathway is helpful in identifying and evaluating present gaps in our knowledge. A well-curated PGDB like the new cattle PGDB developed in this study will facilitate computational reconstruction of metabolic pathways for other mammalian genomes with greater reliability. In addition, it is suggested that an integrated database like the cattle PGDB needs to be recurrently revised as genome annotations and biological databases are updated, and it is important to follow up the current knowledge when conducting a functional genomics study. Like the previous version (i.e., CattleCyc 1.2.1), the new cattle PGDB does not include Y-chromosome specific genes and metabolic pathways because the genome assembly UMD_3.1 does not include Y chromosome. Further work will be required to include the male-specific metabolic pathways by integrating genome annotations with bovine Y chromosome. Also, integrating the information of metabolites to metabolic networks may be a necessary step to improve the quality of metabolic networks and their application. This would be done in a future research on constructing an in silico simulation model of bovine metabolism based on the new CattleCyc developed in this study.

### Identification of gene duplication in cattle

Gene duplication is one of the most important ways to create new genes during a course of evolution. Gene gain can occur either on a large scale from a whole genome duplication or on small scale when chromosomal sections or individual genes are copied. Gene gain is frequently followed by differential gene loss either by mutation in one gene isoform leading to the creation of a pseudogene or by complete deletion. Gene deletion is believed to closely follow duplication in many cases [27]. In the human genome, it has been shown that the whole genome sequences are comprised of up to 5% duplicate sequences [28], and gene duplication is significantly associated with segmental duplication and chromosomal rearrangement [29].

This study identified a total of eight duplicated genes in 12 metabolic pathways of cattle compared to human and mouse. The duplicated genes can be classified according to their possible mechanism, such as segmental duplication and chromosomal rearrangement. An example for each mechanism is given here.

Segmental duplication is one of the possible outcomes of ‘unequal crossing over’, which results from homologous recombination between paralogous sequences [30]. Duplicated blocks of genomic DNA typically range in size from 1–200 kb [31]. Among the duplicated cattle genes, duplication of *AANAT* gene (Chr19: 55,904,213–55,905,449; Chr19: 55,917,031–55,918,872) was due to a segmental duplication in cattle genome. The biological role of vertebrate *AANAT* is to acetylate serotonin in the synthesis of melatonin. It was reported that melatonin promotes sleep, inhibits reproduction, and promotes weight gain [32]. Vertebrate *AANAT* is associated with biological timing: daily changes in the activity of this enzyme regulate the daily rhythm in melatonin synthesis, which is essential for optimal temporal coordination of biological functions with day and night and seasonal changes [33]. Vertebrate *AANAT* is known to be consistently expressed at significant levels only in two tissues, the pineal gland and retina; both of which are photosensitive organs [33–35]. In cattle, it has been reported that SNP in *AANAT* was associated with carcass traits [36, 37].

On the other hand, the duplication of the *SOD1* gene shows an example of outcomes from chromosomal rearrangement. Chromosomal rearrangement by ectopic pairing and recombination between interspersed repeat sequences presents a mechanism for dramatic reorganization of eukaryotic genomes. The *SOD1* gene is located on chr1: 3,113,948–3,122,613 and chr13: 51,930,067–51,930,888 in the cattle genome. The major function of *SOD1* is destruction of free superoxide radicals in the body. The superoxide radical (O_2_^-^) is generated in biological systems as by-product of the partial reduction of dioxygen during respiration. Cytoplasmic Cu, Zn superoxide dismutase catalyzes the dismutation of two superoxide anions into dioxygen and hydrogen peroxide and thus plays a central role in the cellular defense against oxidative stress [38]. Cytosolic Cu, Zn superoxide dismutase, *SOD1*, is a critical component of the cellular defenses against reactive oxygen species and catalyzes the dismutation reaction of the superoxide radical anion to hydrogen peroxide and oxygen via the cycle reduction and re-oxidation of copper [39]. In a study in cattle, Guillemin, Bonnet [40] reported that the *SOD1* gene had a positive correlation with meat tenderness in s*emitendinosus* muscle.

Duplication of the *BPGM* gene can be classified as either chromosomal arrangement or ‘retrotransposition’. Retrotransposition is the integration of reverse transcribed mature RNAs at random sites in a genome. The duplicated gene due to retrotransposition is called as a retrogene, which lacks introns and has poly-A tails [41]. The cattle *BPGM* on chromosome 10 also lacks introns and has a cleavage and polyadenylation specificity factor (CPSF) site and poly-A. The Bisphosphoglycerate mutase, encoded by *BPGM*, is an erythrocyte-specific trifunctional enzyme. The main activity is a synthase (EC 5.4.2.4), catalyzing the formation of 2, 3-bisphosphoglycerate (2,3-BPG) from 1, 3-bisphosphoglycerate (1,3-BPG). The second activity is a mutase (phosphoglycerate mutase, EC 5.4.2.1) catalyzing the interconversion between 2- and 3-phosphoglycerate. The third activity, as a phosphatase (bisphosphoglycerate phosphatase, EC 3.1.3.13), is to catalyze the hydrolysis of 2,3-BPG to 3- or 2-phosphoglycerate and a phosphate [42]. Since the major role of 2,3-BPG is to regulate blood oxygen transport [43, 44], *BPGM* has been studied mostly in erythrocytes and placental cells [42, 43, 45, 46] of human and mouse. In cattle, it was reported that *BPGM* was related with growth rate based on QTL association study [47].

In addition, a phylogenetic analysis was conducted to find more evidence to evolutionarily characterize the duplications of the eight genes. Duplication of *AANAT* was found only in the cattle genome (S1 Fig), whereas duplication of the other genes was observed in other mammalian genomes except the human and mouse genomes. For example, *SOD1* is duplicated in African savanna elephant and triplicated in small-eared galago (S2 Fig). *BPGM* is also duplicated in white-tufted-ear marmoset and European shrew (S3 Fig). There is thus a possibility that some of these gene duplications may be a neutral event and link to evolutionary adaptations in ecological contexts. Nevertheless, it is not conclusive whether these duplications actually occurred in other mammalian genomes due to their low sequence coverage genome (~2x) and incomplete annotations. One needs to be cautious when conducting and interpreting a phylogenetic analysis for gene evolution. The genomes with low quality sequences and incomplete annotations contain falsely predicted genes, and they can introduce bias in a phylogenetic analysis [48, 49].

Therefore, even though the eight genes duplicated in the cattle genome are apparently absent in the human and mouse genomes, it is not clearly understood whether these gene are cattle-specific gene gain or gene losses in the human and mouse genomes and what the biological significance of these duplications is. This study focused more on genome-wide structural variations in the metabolic genes and discussion of possible biological consequences. More researches need to be conducted to validate and investigate experimental evidence of the duplications found in this study. The biological functions and advantage of these duplications in cattle also need to be elucidated.

## Conclusions

In this study, we developed a bioinformatic pipeline for integrating all the information for genes and proteins from multiple sources including NCBI, Ensembl, UniProt, and KEGG and constructing an amalgamated genome database of an organism. Cattle-specific metabolic pathways were automatically reconstructed by comparing the amalgamated cattle genome database and well-curated metabolic database, MetaCyc, followed by an intensive manual curation. We found the automated reconstruction using the PathoLogic algorithm in Pathway Tools still contains errors such as missing enzymes and metabolic pathways, so that an intensive manual curation is unavoidable. We also found there has been a significant improvement in the quality of cattle genome annotations and MetaCyc.

This study found duplicated metabolic genes in the cattle genome compared to the human and mouse genomes, which implies evolutionary changes in the cattle genome and provides a possible explanation for metabolic adaptation of cattle.

The updated genome-scale metabolic reconstruction from this study will provide a helpful tool to further studies for understanding underlying mechanism of biological function and metabolic traits in cattle. Further research needs to be conducted to validate the results found in this study by experimental evidences and to elucidate the biological functions and advantage of these duplications in cattle.

## Supporting Information

S1 Fig Phylogenetic tree for *AANAT* gene of cattle genome compared to the protein sequence of mammals. The phylogenetic tree was generated for putative duplicated genes using MEGA 6.0 [50]. The protein sequence of each genes in mammals was downloaded from Ensemble. Then, the protein sequence of each mammals was aligned and removed for the ambiguous position using MUSCLE algorithm [51]. The bootstrap consensus tree with 1,000 replicates was constructed using the Neighbor-Joining method [52] based on the JTT matrix [53] based model conducted.
(EPS)

S2 Fig Phylogenetic tree for *SOD1* gene of cattle genome compared to the protein sequence of mammals. The phylogenetic tree was generated for putative duplicated genes using MEGA 6.0 [50]. The protein sequence of each genes in mammals was downloaded from Ensemble. Then, the protein sequence of each mammals was aligned and removed for the ambiguous position using MUSCLE algorithm [51]. The bootstrap consensus tree with 1,000 replicates was constructed using the Neighbor-Joining method [52] based on the JTT matrix [53] based model conducted.
(EPS)

S3 Fig Phylogenetic tree for *BPGM* gene of cattle genome compared to the protein sequence of mammals. The phylogenetic tree was generated for putative duplicated genes using MEGA 6.0 [50]. The protein sequence of each genes in mammals was downloaded from Ensemble. Then, the protein sequence of each mammals was aligned and removed for the ambiguous position using MUSCLE algorithm [51]. The bootstrap consensus tree with 1,000 replicates was constructed using the Neighbor-Joining method [52] based on the JTT matrix [53] based model conducted.
(EPS)

S1 Table List of manually curated pathways in the new cattle-specific pathway genome database (PGDB) (DOCX)

S2 Table Comparison of the pathways in the new cattle-specific pathway genome database (PGDB) based on UMD_3.1 with those in the previous version of cattle PGDB based on Btau_3.1 (CattleCyc 1.2.1; http://168.188.16.73:8080/CATTLE) (DOCX)
