# Supplementary material for: Global Metabolic Reconstruction and Metabolic Gene Evolution in the Cattle Genome
Source: PLoS One. 2016 Mar 18;11(3):e0150974. doi: 10.1371/journal.pone.0150974 (PMC4798299; doi:10.1371/journal.pone.0150974)
Supplement: S1 Table — (DOCX) [file pone.0150974.s004.docx]

**S1 Table. List of manually curated pathways in the new cattle-specific pathway genome database (PGDB)**

| Pathway | Type | Comment |
| --- | --- | --- |
| 2-methylbutyrate biosynthesis | Deleted | A redundant pathway with 2-methylbutyrate biosynthesis mammals |
| 4-hydroxyproline degradation I | Deleted | A redundant pathway with 4-hydroxyproline degradation I mammals |
| arginine biosynthesis IV | Deleted | A redundant pathway with arginine biosynthesis IV mammals |
| gluconeogenesis I | Deleted | A redundant pathway with gluconeogenesis mammals |
| glutaryl-CoA degradation | Deleted | A redundant pathway with glutaryl-CoA degradation mammals |
| glycolysis I | Deleted | A redundant pathway with glycolysis mammals |
| isoleucine degradation I | Deleted | A redundant pathway with isoleucine degradation I mammals |
| leucine degradation I | Deleted | A redundant pathway with leucine degradation I mammals |
| salvage pathways of pyrimidine deoxyribonucleotides | Deleted | A redundant pathway with salvage pathways of pyrimidine deoxyribonucleotides mammals |
| salvage pathways of pyrimidine ribonucleotides | Deleted | A redundant pathway with salvage pathways of pyrimidine ribonucleotides mammals |
| valine degradation I | Deleted | A redundant pathway with valine degradation I mammals |
| valine biosynthesis | Deleted | Non-mammalian pathway |
| L-Nδ-acetylornithine biosynthesis | Deleted | Non-mammalian pathway |
| phenylethanol biosynthesis | Deleted | Non-mammalian pathway |
| chitin degradation II | Deleted | Non-mammalian pathway |
| glucose and glucose-1-phosphate degradation | Deleted | Non-mammalian pathway |
| isoleucine biosynthesis I (from threonine) | Deleted | Non-mammalian pathway |
| lysine degradation V | Deleted | Non-mammalian pathway |
| CMP-N-acetylneuraminate biosynthesis II (bacteria) | Deleted | Non-mammalian pathway |
| D-serine degradation | Deleted | Non-mammalian pathway |
| melibiose degradation | Deleted | Non-mammalian pathway |
| methyl parathion degradation | Deleted | Non-mammalian pathway |
| phosphate acquisition | Deleted | Non-mammalian pathway |
| phosphate utilization in cell wall regeneration | Deleted | Non-mammalian pathway |
| TCA cycle I (prokaryotic) | Deleted | Non-mammalian pathway |
| wound-induced proteolysis I | Deleted | Non-mammalian pathway |
| camptothecin biosynthesis | Deleted | Non-mammalian pathway |
| carotenoid cleavage dioxygenases | Deleted | Non-mammalian pathway |
| acidification and chitin degradation (in carnivorous plants) | Deleted | Non-mammalian pathway |
| seed germination protein turnover | Deleted | Non-mammalian pathway |
| formaldehyde oxidation IV (thiol-independent) | Deleted | Non-mammalian pathway |
| glycine betaine biosynthesis I (Gram-negative bacteria) | Deleted | Non-mammalian pathway |
| glycine betaine biosynthesis II (Gram-positive bacteria) | Deleted | Non-mammalian pathway |
| glycine betaine biosynthesis V (from glycine) | Deleted | Non-mammalian pathway |
| fatty acids biosynthesis (yeast) | Deleted | Non-mammalian pathway |
| tRNA methylation (yeast) | Deleted | Non-mammalian pathway |
| 1D-myo-inositol hexakisphosphate biosynthesis II (mammalian) | Modified | EC 2.7.1.140 is annotated with 615135 by manual curation |
| 2-amino-3-carboxymuconate semialdehyde degradation to glutaryl-CoA | Modified | The enzymatic reaction complex with EC1.2.4.2 and EC 2.3.1.61 was created by manual curation. |
| acetone degradation I (to methylglyoxal) | Modified | EC 4.1.1.4 is replaced by spontaneous reaction by manual curation |
| androgen biosynthesis | Modified | EC 4.1.2.30 is annotated with 281739 by manual curation |
| arginine degradation I (arginase pathway) | Modified | EC 1.5.1.12, a reaction of 2013, is replaced by EC 1.2.1.8 by manual curation |
| arsenate detoxification I (glutaredoxin) | Modified | EC 1.20.4.2 is annotated with 613905,785216 and 505642 by manual curation |
| bile acid biosynthesis, neutral pathway | Modified | EC 1.1.1.161, 1.2.1.40 deleted entry on 2012 and it replace to EC 1.14.13.15. Also, EC 1.1.1.213, EC 1.17.99.3 and EC 6.2.1.7 are annotated with 100300732, 514969 and 533016, respectively. In addition, EC 1.1.1.35 encoded to protein from 281809, 493643, 518852 and 532785 by manual curation. |
| cardiolipin biosynthesis II | Modified | EC 3.1.3.27 is annotated with 614890 by manual curation |
| CDP-diacylglycerol biosynthesis I | Modified | EC 1.1.1.8 is annotated with 514456 and 525042 by manual curation |
| ceramide biosynthesis | Modified | EC2.3.1.24 is annotated with 100126279, 100127167, 100152265, 100156737, 100525450, 100626198 by manual curation |
| ceramide degradation | Modified | EC 2.7.1.91 is annotated with 533103 and 618605 by manual curation |
| chondroitin sulfate biosynthesis (late stages) | Modified | EC 2.8.2.- is annotated with 535975 by manual curation |
| degradation of purine deoxyribonucleosides mammals | Modified | EC 3.5.4.4 is annotated with 280712 and 617805 by manual curation |
| dermatan sulfate biosynthesis (late stages) | Modified | EC 2.8.2.- is annotated with 535975 by manual curation |
| D-myo-inositol (1,4,5,6)-tetrakisphosphate biosynthesis | Modified | EC 2.7.1.140 is annotated with 615135 by manual curation |
| D-myo-inositol (3,4,5,6)-tetrakisphosphate biosynthesis | Modified | EC 2.7.1.140 is annotated with 615135 by manual curation |
| eumelanin biosynthesis | Modified | EC 1.14.18.1 is annotated with 280951 by manual curation |
| fatty acid beta-oxidation V (unsaturated, odd number, di-isomerase-dependent) | Modified | EC 5.3.3.- is annotated with 507431 by manual curation |
| folate transformations I | Modified | Non-mammalian enzymes, EC 2.1.1.19 and EC 1.5.7.1, were deleted due to |
| formylTHF biosynthesis I | Modified | EC 6.3.2.17 is annotated with 505809 by manual curation |
| glutaryl-CoA degradation mammals | Modified | EC 4.2.1.55 is annotated with 281748, 281810 and 518852. Also, EC 1.1.1.35 encoded to protein from 281809, 493643, 518852 and 532785 by manual curation |
| glutathione-mediated detoxification | Modified | EC 4.4.1.13 is annotated with 508172 and 528582 by manual curation |
| histamine degradation | Modified | Non-mammal's enzyme, EC 6.3.2.18 deleted due to |
| isoleucine degradation I mammals | Modified | The complex enzymatic reactions create to use EC1.2.4.4, EC 1.8.1.4, and EC 2.3.1.168. In addition, EC 1.3.8.1 is annotated with 511222 by manual curation |
| ketogenesis | Modified | EC 4.1.1.4 replace to spontaneous reaction by manual curation |
| L-ascorbate biosynthesis VI | Modified | EC 2.4.1.17 is annotated with 100138004, 100138908, 100140261, 100296421, 286792, 511743, 515638, 530553, 533587, 540615, 615303 and 751790 by manual curation |
| leucine degradation I mammals | Modified | The complex enzymatic reactions create to use EC1.2.4.4, EC 1.8.1.4, and EC 2.3.1.168. Also, the EC 1.3.8.1 is annotated with 511222 by manual curation |
| L-glutamine biosynthesis II (tRNA-dependent) | Modified | EC 6.1.1.24 is annotated with 100300732 by manual curation |
| lysine degradation II | Modified | The complex enzymatic reactions create to use EC1.2.4.2 and EC 2.3.1.61 by manual curation |
| lysine degradation VII mammals | Modified | EC 1.5.3.7 is annotated with 509102 by manual curation |
| methionine degradation I (to homocysteine) | Modified | EC 2.1.1.37 is annotated with 100848258, 100848415, 28119, 353354, 359716 and 613785 by manual curation |
| methylglyoxal degradation I | Modified | EC 1.1.2.4 is annotated with 510284 by manual curation |
| molybdenum cofactor biosynthesis | Modified | EC 2.8.1.11 is annotated with 539728 by manual curation |
| NAD biosynthesis I (from aspartate) | Modified | Non-mammal’s enzyme, EC 6.3.1.5 deleted due to. In addition, EC 1.4.3.16 replace to EC 1.4.1.21 |
| NADH repair | Modified | EC 5.1.99.6 is annotated with 404132 by manual curation |
| oleate biosynthesis II (animals) | Modified | EC 3.1.2.2 is annotated with 511033, 511431, 512541, 514788, 530653 and 76798 by manual curation. |
| palmitate biosynthesis I (animals) | Modified | The EC 4.2.1.58 deleted entry on 2012 and it replace to EC 4.2.1.59. EC 1.3.1.39 and EC 4.2.1.59 is annotated with 281152. In addition, EC 3.1.2.21 is annotated with 617347 by manual curation |
| phenylalanine degradation IV (mammalian, via side chain) | Modified | EC 4.1.1.53 and EC 1.2.1.39 replace to 4.1.1.28 and 1.2.1.5, respectively. Also, EC 1.4.3.21 is annotated with 281002, 789307, 100138645 and 100336082 by manual curation |
| phenylethylamine degradation I | Modified | EC 1.2.1.5 is annotated with 281617,507093,508879 and 511469 by manual curation |
| pregnenolone biosynthesis | Modified | EC 1.14.13.- replace to EC 1.14.15.6 by manual curation |
| proline biosynthesis II (from arginine) | Modified | EC 3.5.3.6 replace to EC 1.14.13.39 by manual curation |
| proline degradation | Modified | EC 1.5.1.12 deleted entry on 2013 and it replaced to EC 1.2.1.88 |
| pyridine nucleotide cycling mammals | Modified | EC 2.7.1.1 is annotated with 100337285, 511042 and 522863 by manual curation |
| pyridoxal 5'-phosphate salvage pathway | Modified | EC 2.7.1.- replace to EC 2.7.1.35 by manual curation |
| retinoate biosynthesis I | Modified | The unassigned EC number for biosynthesis of retinol encoded to protein 281444 by manual curation |
| salvage pathways of pyrimidine ribonucleotides mammals | Modified | EC 2.7.1.48 is annotated with 519697, 534046 and 541028 by manual curation |
| spermine and spermidine degradation I | Modified | EC 2.3.1.57 is annotated with 359722 and 508861 by manual curation |
| sphingolipid metabolism mammals | Modified | EC 3.1.3.4 is annotated with 504545, 617172 and 617707 as well as EC 3.1.3.- is 100337041 and 516881. Also, EC 2.4.1.274 and EC 3.1.6.1 are annotated with 524460 and 505899, respectively, by manual curation |
| sphingosine and sphingosine-1-phosphate metabolism | Modified | EC 4.1.2.27 is annotated with 522515 by manual curation |
| triacylglycerol biosynthesis | Modified | Non-mammal's enzyme, EC 2.3.1.158 deleted due to |
| triacylglycerol degradation | Modified | Change EC 3.1.1.34 to EC 3.1.1.79, and it is annotated with 286879 (LIPE), and EC 3.1.1.- replace to 3.1.1.3 by manual curation |
| ubiquinol-10 biosynthesis (eukaryotic) | Modified | EC 1.14.13.and EC 1.14.-.- are annotated with COQ6 and COQ7 by manual curation |
| ubiquinol-9 biosynthesis (eukaryotic) | Modified | EC 1.14.13.and EC 1.14.-.- are annotated with COQ6 and COQ7 by manual curation |
| valine degradation I mammals | Modified | The complex enzymatic reactions create to use EC1.2.4.4, EC 1.8.1.4, and EC 2.3.1.168. Also, EC 1.3.8.4 is annotated with 510440 and EC 4.2.1.17 encoded to protein from 281748, 281810, and 518852 by manual curation |
| xanthine and xanthosine salvage | Modified | EC 2.4.2.22 replace to EC 2.4.2.8 by manual curation |
| β-alanine degradation I | Modified | EC 1.2.1.18 changed in final product to acetyl-CoA by manual curation |
| γ-linolenate biosynthesis II (animals) | Modified | EC 1.2.1.18 is annotated with 521822 and 533107 by manual curation |
