# Supplementary material for: Global Metabolic Reconstruction and Metabolic Gene Evolution in the Cattle Genome
Source: PLoS One. 2016 Mar 18;11(3):e0150974. doi: 10.1371/journal.pone.0150974 (PMC4798299; doi:10.1371/journal.pone.0150974)
Supplement: S2 Table — (DOCX) [file pone.0150974.s005.docx]

S2 Table. Comparison of the pathways in the new cattle-specific pathway genome database (PGDB) based on UMD_3.1 with those in the previous version of cattle PGDB based on Btau_3.1 (CattleCyc 1.2.1; http://168.188.16.73:8080/CATTLE)

| Pathway | Comment |
| --- | --- |
| sphingolipid metabolism mammals | Added to UMD_3.1 |
| starch degradation mammals | Added to UMD_3.1 |
| sucrose degradation mammals | Added to UMD_3.1 |
| UDP-D-glucuronate biosynthesis (from myo-inositol) mammals | Added to UMD_3.1 |
| UDP-glucose conversion | Added to UMD_3.1 |
| myo-inositol biosynthesis | Added to UMD_3.1 |
| N-acetylneuraminate degradation mammals | Added to UMD_3.1 |
| octane oxidation mammals | Added to UMD_3.1 |
| proline biosynthesis III | Added to UMD_3.1 |
| putrescine degradation IV | Added to UMD_3.1 |
| pyridine nucleotide cycling mammals | Added to UMD_3.1 |
| lysine degradation VII mammals | Added to UMD_3.1 |
| mannose degradation mammals | Added to UMD_3.1 |
| fatty acid ω-oxidation | Added to UMD_3.1 |
| glutamine degradation II mammals | Added to UMD_3.1 |
| glycine betaine biosynthesis (from glycine) mammals | Added to UMD_3.1 |
| glycine degradation I | Added to UMD_3.1 |
| degradation of purine deoxyribonucleosides mammals | Added to UMD_3.1 |
| degradation of pyrimidine deoxyribonucleosides mammals | Added to UMD_3.1 |
| fatty acid biosynthesis initiation I | Added to UMD_3.1 |
| fatty acid biosynthesis initiation III | Added to UMD_3.1 |
| (deoxy)ribose phosphate degradation mammals | Added to UMD_3.1 |
| arginine degradation I (arginase pathway) | Added to UMD_3.1 |
| 4-aminobutyrate degradation II | Due to the MetaCyc update |
| acetyl-CoA biosynthesis (from citrate) | Due to the MetaCyc update |
| acetyl-CoA biosynthesis (from pyruvate) | Due to the MetaCyc update |
| aerobic respiration -- electron donor II | Due to the MetaCyc update |
| ascorbate biosynthesis mammals | Due to the MetaCyc update |
| asparagine degradation II | Due to the MetaCyc update |
| aspartate degradation I | Due to the MetaCyc update |
| citrulline degradation mammals | Due to the MetaCyc update |
| cysteine biosynthesis II | Due to the MetaCyc update |
| de novo biosynthesis of pyrimidine deoxyribonucleotides mammals | Due to the MetaCyc update |
| fatty acid α-oxidation mammals | Due to the MetaCyc update |
| fatty acid β-oxidation IV (unsaturated, even number) | Due to the MetaCyc update |
| folate polyglutamylation I | Due to the MetaCyc update |
| folate polyglutamylation II | Due to the MetaCyc update |
| folate transformations mammals | Due to the MetaCyc update |
| formylTHF biosynthesis II mammals | Due to the MetaCyc update |
| glutamate degradation IV mammals | Due to the MetaCyc update |
| glutamine biosynthesis II | Due to the MetaCyc update |
| glycerol degradation IV mammals | Due to the MetaCyc update |
| glycine betaine biosynthesis (from choline) mammals | Due to the MetaCyc update |
| histidine degradation III mammals | Due to the MetaCyc update |
| isoleucine degradation I mammals | Due to the MetaCyc update |
| Ketone oxidation | Due to the MetaCyc update |
| methylglyoxal degradation mammals | Due to the MetaCyc update |
| NAD biosynthesis I (from aspartate) mammals | Due to the MetaCyc update |
| NAD biosynthesis II (from tryptophan) mammals | Due to the MetaCyc update |
| NAD salvage pathway II mammals | Due to the MetaCyc update |
| NAD/NADH phosphorylation and dephosphorylation mammals | Due to the MetaCyc update |
| oxidative ethanol degradation mammals | Due to the MetaCyc update |
| parathion degradation | Due to the MetaCyc update |
| proline biosynthesis II | Due to the MetaCyc update |
| proline biosynthesis V (from arginine) mammals | Due to the MetaCyc update |
| proline degradation II | Due to the MetaCyc update |
| purine degradation mammals | Due to the MetaCyc update |
| purine nucleotides de novo biosynthesis I mammals | Due to the MetaCyc update |
| purine ribonucleosides degradation | Due to the MetaCyc update |
| putrescine biosynthesis I | Due to the MetaCyc update |
| putrescine biosynthesis III | Due to the MetaCyc update |
| pyruvate degradation II mammals | Due to the MetaCyc update |
| riboflavin metabolism mammals | Due to the MetaCyc update |
| S-adenosyl-L-methionine cycle mammals | Due to the MetaCyc update |
| S-adenosylmethionine biosynthesis mammals | Due to the MetaCyc update |
| TCA cycle mammals | Due to the MetaCyc update |
| trans, trans-farnesyl diphosphate biosynthesis I | Due to the MetaCyc update |
| tRNA charging pathway | Due to the MetaCyc update |
| tryptophan degradation I (via anthranilate) mammals | Due to the MetaCyc update |
| tryptophan degradation III (eukaryotic) mammals | Due to the MetaCyc update |
| tryptophan degradation VI (via tryptamine) mammals | Due to the MetaCyc update |
| tyrosine biosynthesis mammals | Due to the MetaCyc update |
| UDP-galactose biosynthesis (salvage pathway from galactose using UDP-glucose) | Due to the MetaCyc update |
| UDP-N-acetyl-D-glucosamine biosynthesis mammals | Due to the MetaCyc update |
| uracil degradation (reductive) | Due to the MetaCyc update |
| β-alanine degradation I | Due to the MetaCyc update |
| aerobic respiration -- electron donors reaction list | Insufficient evidence of its incorporate in the new cattle PGDB |
| aspartate biosynthesis II | Insufficient evidence of its incorporate in the new cattle PGDB |
| galactose assimilation III mammals | Insufficient evidence of its incorporate in the new cattle PGDB |
| isoleucine degradation II | Insufficient evidence of its incorporate in the new cattle PGDB |
| lipoate biosynthesis and incorporation II | Insufficient evidence of its incorporate in the new cattle PGDB |
| phospholipid biosynthesis II mammals | Insufficient evidence of its incorporate in the new cattle PGDB |
| ribose degradation | Insufficient evidence of its incorporate in the new cattle PGDB |
| salvage pathways of adenine, hypoxanthine, and their nucleosides mammals | Insufficient evidence of its incorporate in the new cattle PGDB |
| salvage pathways of guanine, xanthine, and their nucleosides mammals | Insufficient evidence of its incorporate in the new cattle PGDB |
| salvage pathways of purine and pyrimidine nucleotides mammals | Insufficient evidence of its incorporate in the new cattle PGDB |
| salvage pathways of purine nucleosides mammals | Insufficient evidence of its incorporate in the new cattle PGDB |
| tetrahydrofolate biosynthesis I mammals | Insufficient evidence of its incorporate in the new cattle PGDB |
| tetrahydrofolate biosynthesis II mammals | Insufficient evidence of its incorporate in the new cattle PGDB |
| β-alanine biosynthesis II mammals | Insufficient evidence of its incorporate in the new cattle PGDB |
| actinorhodin biosynthesis | Non mammalian pathway included in the previous version |
| alanine degradation II (to D-lactate) | Non mammalian pathway included in the previous version |
| aminopropylcadaverine biosynthesis | Non mammalian pathway included in the previous version |
| ascorbate glutathione cycle | Non mammalian pathway included in the previous version |
| asparagine biosynthesis II | Non mammalian pathway included in the previous version |
| carnitine degradation II | Non mammalian pathway included in the previous version |
| carnitine degradation III | Non mammalian pathway included in the previous version |
| choline biosynthesis I | Non mammalian pathway included in the previous version |
| fatty acid elongation -- saturated | Non mammalian pathway included in the previous version |
| isoleucine biosynthesis V | Non mammalian pathway included in the previous version |
| leucine degradation III | Non mammalian pathway included in the previous version |
| methylerythritol phosphate pathway | Non mammalian pathway included in the previous version |
| pyridoxamine anabolism | Non mammalian pathway included in the previous version |
| selenocysteine biosynthesis I (bacteria) | Non mammalian pathway included in the previous version |
| valine degradation II | Non mammalian pathway included in the previous version |
| xylitol degradation | Non mammalian pathway included in the previous version |
